# Maternal mortality: a cross-sectional study in global health

**DOI:** 10.1186/s12992-015-0087-y

**Published:** 2015-02-12

**Authors:** Sima Sajedinejad, Reza Majdzadeh, AbouAli Vedadhir, Mahmoud Ghazi Tabatabaei, Kazem Mohammad

**Affiliations:** Department of Epidemiology and Biostatistics, School of Public Health, Tehran University of Medical Sciences (TUMS), Tehran, Iran; Knowledge Utilization Research Center (KURC), Tehran University of Medical Sciences (TUMS), Tehran, Iran; Department of Anthropology, Faculty of Social Sciences, University of Tehran (UT), Tehran, Iran; Department of Demography and Population Studies, Faculty of Social Sciences, University of Tehran, Tehran, Iran

**Keywords:** Maternal mortality, Global data, Macrostructural indicators, Governance, Education, Post-2015 development

## Abstract

**Background:**

Although most of maternal deaths are preventable, maternal mortality reduction programs have not been completely successful. As targeting individuals alone does not seem to be an effective strategy to reduce maternal mortality (Millennium Development Goal 5), the present study sought to reveal the role of many distant macrostructural factors affecting maternal mortality at the global level.

**Methods:**

After preparing a global dataset, 439 indicators were selected from nearly 1800 indicators based on their relevance and the application of proper inclusion and exclusion criteria. Then Pearson correlation coefficients were computed to assess the relationship between these indicators and maternal mortality. Only indicators with statistically significant correlation more than 0.2, and missing values less than 20% were maintained. Due to the high multicollinearity among the remaining indicators, after missing values analysis and imputation, factor analysis was performed with principal component analysis as the method of extraction. Ten factors were finally extracted and entered into a multiple regression analysis.

**Results:**

The findings of this study not only consolidated the results of earlier studies about maternal mortality, but also added new evidence. Education (std. B = −0.442), private sector and trade (std. B = −0.316), and governance (std. B = −0.280) were found to be the most important macrostructural factors associated with maternal mortality. Employment and labor structure, economic policy and debt, agriculture and food production, private sector infrastructure investment, and health finance were also some other critical factors. These distal factors explained about 65% of the variability in maternal mortality between different countries.

**Conclusion:**

Decreasing maternal mortality requires dealing with various factors other than individual determinants including political will, reallocation of national resources (especially health resources) in the governmental sector, education, attention to the expansion of the private sector trade and improving spectrums of governance. In other words, sustainable reduction in maternal mortality (as a development indicator) will depend on long-term planning for multi-faceted development. Moreover, trade, debt, political stability, and strength of legal rights can be affected by elements outside the borders of countries and global determinants. These findings are believed to be beneficial for sustainable development in Post-2015 Development Agenda.

**Electronic supplementary material:**

The online version of this article (doi:10.1186/s12992-015-0087-y) contains supplementary material, which is available to authorized users.

## Background

Some health indicators are known to reflect not only as the overall status of the health care system, but also various aspects of a country’s structure. Maternal mortality is widely accepted as a key indicator of health and socioeconomic development [1]. It is a reflection of the whole national health system and represents the outcome of its cons and pros along with its other characteristics such as intersectoral collaboration, transparency and disparities. Beyond these, it can also illustrate even the sociocultural, political and economic philosophy of a society.

Improving maternal health and reducing maternal mortality ratio (MMR) by 75% between 1990 and 2015 have been defined as the Millennium Development Goal 5 (MDG 5A) [2]. Achieving all MDGs is still a major challenge to the health systems worldwide [3]. Despite the fact that most maternal deaths are preventable, progress in controlling such deaths has not been satisfactory [4]. Therefore, the MDGs cannot be successfully practiced due to data gaps, inconsistent indicators, and frequent revisions [5].

The global MMR reduced from 380 maternal deaths per 100 000 live births in 1990 to 210 maternal deaths per 100 000 live births in 2010 [6]. Moreover, in 2013, the rate was 45% lower than that in 1990. Sub-Saharan Africa and Southern Asia are believed to account for 62% and 24% of global deaths, respectively. On the other hand, one third of all maternal deaths have been found to occur in India (17%) and Nigeria (14%). While the MMR in developing regions is 15 times higher than that in developed regions (230 vs. 15), the greatest MMR, 510 maternal deaths per 100 000 live births, has been reported from Sub-Saharan Africa. Belarus, Maldives, and Bhutan had the largest declines in MMR between 1990 and 2013 [6].

A systematic review in 2006 reviewed studies on maternal mortality published during 2000–2004 and revealed that researchers mainly focused on clinical aspects of the problem rather than the contributing sociocultural, economic, and political factors. It also suggested that research on maternal mortality suffered from robust methodological design to produce knowledge about macrostructural causes of maternal mortality [7]. Although health care plays a critical role in maternal mortalities, the effects of other factors, e.g. female education and accessibility to health facilities, should not be neglected [8]. However, the reasons for higher declines in MMR in some countries and the absence of progress in some others have not been fully discovered [9]. Although maternal mortality is extensively recognized as a main indicator of health and socioeconomic development [1], evidence for such association is limited.

As the above-mentioned systematic review highlighted the need for knowledge about the macrostructural causes of maternal mortality [7], the present study investigated the relationship between some macrostructural factors and maternal mortality at the global level in 2010. In other words, it sought to determine the impact of development on maternal mortality.

An ecological study generally compares groups rather than individuals [10]. Ecological variables can be classified in various categories [11,12]. While some variables are expressed as median, mean, or sometimes standard deviation of individual indicators like percentage of school enrollment, some others cannot be measured at the individual level and have a figure for a country or region (e.g. government effectiveness). An important point about ecological studies is attention to the level of data aggregation and inference. Cross-level inference while ignoring inter-area and between-area variability, can sometimes result in ecological bias [10,11].

## Methods

This cross-sectional study was conducted on 2010 ecological data from 179 countries. The studied indicators either were aggregated (e.g. labor participation rate) or had a single measure for each country (e.g. rule of law).

### Data source

A- Maternal mortality
  Outlining the trend of maternal mortality has concerned many scholars in recent years [1,6,9,13-15]. We adopted the methodology described by Wilmoth et al. [1] and selected the reports of the World Health Organization (WHO), United Nations International Children’s Emergency Fund (UNICEF), United Nations Population Fund (UNFPA), and the World Bank [9] to collect MMR data for 2010. The 181 countries and territories included in this report represented 99.9% of global births. In total, these countries (or territories) were divided into three categories based on the underlying data used to generate the country-specific estimates: (A) countries with relatively complete civil registration systems and good attribution of causes of death; (B) countries without perfect maternal mortality-related data registration, but with other types of data available; and (C) countries with no available national-level data on maternal mortality [9]. It is noteworthy that only 4% of births took place in group C countries/territories.
  For group A, vital registration information was directly applied to estimate MMR. For countries in groups B and C, a two-part multilevel regression model was developed using national-level data from various sources such as civil registration, surveys, surveillance systems, censuses, reproductive age mortality studies, and sample registration systems. Afterward, the proportion of acquired immune deficiency syndrome (AIDS) deaths that qualified as indirect maternal deaths to the total number of AIDS deaths among women aged 15–49 years was calculated. The three selected predictor variables in the regression model were gross domestic product (GDP), general fertility rate (GFR), and presence of a skilled attendant at birth (SAB) as a proportion of live births. These predictor variables were chosen from a broader list of potential predictor variables comprising indicators of socioeconomic development (such as GDP, human development index, and female life expectancy at birth), process variables (e.g. SAB, proportions receiving antenatal care, proportion of institutional births), and risk exposure as a function of fertility (GFR or the total fertility rate) [1]. This methodology was important for the including criteria to select proper indicators in the next steps.
B- Other global indicators
  The World Bank database [16] consists of 55 databanks in 20 topics. We excluded topic- or region-specific databases such as Africa Infrastructure: Electricity, G20 Financial Inclusion Indicators, and India Power Sector. In order to obtain global data, we selected the following databases based on their data availability and relation to our research topic:
  1. *World Development Indicators (WDI)*: It is the primary World Bank collection of development indicators gathered from officially recognized international sources. It presents the most current and accurate global development data available and includes national, regional, and global estimates.
  2. *Education Statistics Database*: It compiles data on education from national statistical reports, statistical annexes of new publications, and other data sources.
  3. *Gender Statistics Database*: It provides data on key gender topics. Included themes are demographics, education, health, labor force, and political participation.
  4. *Health Nutrition and Population Statistics*: Key health, nutrition, and population statistics collected from different international sources.
  5. *Poverty and Inequality Database*
  6. *MDGs*: It is composed of official indicators for monitoring progress toward MDGs.
  7. *Worldwide Governance Indicators*: It provides aggregate and individual governance indicators for six dimensions of governance for 213 economies over the period of 1996–2009.

We also reviewed different sources for other global indicators such as global terrorism index, global peace index, international homicide index, and democracy index and considered the important indicators in this study.

### Data preparation and analysis

I. Indicator selection process
  Database selection: After evaluating all databases, the most relevant ones were selected as explained above.
  Indicator selection criteria: All economic, political, sociocultural, and health system-related indicators with direct or indirect effects were selected from each database if:
  they were adjusted (e.g. percentage or per-capita) to be comparable with other countries;
  they did not either relate to the predictors of MMR in the model (GDP, SAB, and GFR) or the broader list of potential predictor variables (as mentioned earlier in the maternal mortality data section) or have obvious correlations with them like gross national product (GNP). Hence, none of the HIV/AIDS-related indicators was selected since they were used in MMR prediction for some countries; and
  they were not health system outcomes similar to MMR (which were affected by the same distal macrostructural predictors such as infant or child mortality rates).

In case of overlaps between databases, especially among MDGs or gender databases and other groups, repeated indicators were considered only once, preferably in the most relevant group like education, health, or employment.

Since each database covered a particular number of countries, we just selected the countries which were common between the WHO report and the World Bank database. Ultimately, 439 indicators were selected out of more than 1800 reviewed ones.

Major concerns about the selected indicators: Three issues mainly concerned the researchers:

- High number of the indicators
- Probability of multicollinearity, a statistical phenomenon in which more than two independent variables are highly correlated [17-19], among indicators of each category and between categories. Such conditions might prevent statistical significance and enlarge confidence intervals (sometime containing zero).
- Missing values

For most aggregated variables, especially education and employment indicators, e.g. primary school completion rate, three figures were available, i.e. two for females and males and a total value. Based on the research objectives, we excluded the values related to males and worked with the other two values in the next steps.

In order to minimize the missing values, the nearest figure among ± 3 years to 2010 was selected in the absence of an exact value for 2010. If two years with equal distance from 2010 had different values, the average value was considered. For instance, if the figure for 2010 was not available, but the values for both 2009 and 2011 were present, the average was calculated and used.

Bivariate correlation with maternal mortality: In the next step, bivariate correlations between maternal mortality and all of the 439 selected indicators were calculated (Table 1). Data were not available for 22 indicators and there were 1–2 values for four indicators (which did not provide any significance level).

**Table 1:** **Summary of bivariate correlations between MMR and each selected global indicator**

		**Pearson correlation index**									
		0-0.2			0.2- 0.5			0.5- 1.0			Total
Level of significance		≤0.05	0.05-0.2	≥0.2	≤0.05	0.05-0.2	≥0.2	≤0.05	0.05-0.2	≥0.2	
	<30	0	1	9	8	11	2	14	0	1	46
% of data availability	30-50	1	8	21	35	1	0	18	0	0	84
	50-80	2	11	18	51	0	1	45	0	3	131
	>80	16	16	25	*68*	0	0	*52*	0	1	178
	Total	19	36	73	162	12	3	129	0	5	439
		128			177			134			

As bivariate correlations of MMR with indicators having two values for females and the total population did not show any important differences, we could not eliminate any of them. Moreover, in order to decrease the level of uncertainty, we decided to select the indicators with minimum missing values.

In an attempt to select the indicators based on the assessment of bivariate correlations, two scenarios were tested. In the first scenario, indicators with correlations more than 0.5, significance level of less than 0.05, and missing values less than 20% were evaluated. Only 38 indicators from six categories remained. However, no indicators from the major categories (based on the World Bank categories for World Development Indicator Database) including environment, economic policy and external debt, private sector and trade, poverty and inequality, gender, and labor and social protection remained. In the second scenario, we considered indicators with correlations more than 0.2, significance level of less than 0.05, and missing data less than 20%. In this scenario, 116 indicators from seven different categories, in 24 subcategories remained (Additional file 1). The seven main categories were private sector and trade, governance, education (input, outcome, participation, and efficiency), employment and social protection, economic policy and debt, health service expenditure (service), environment-agriculture and production. The second scenario, which could cover better diversity of indicators under each category, was selected for further analyses.

Unfortunately, due to over 50% missing data for all indicators, none of the indicators in the poverty and inequality databas were seen in the selected indicators. On the other hand, since the eligible indicators remaining from the gender database were common with some other groups, like employment and education, we kept them under the main category (Additional file 1). The absolute value of the correlation in this scenario ranged from 0.201 to 0.871.

Missing value imputation: As described above, we excluded variables with missing values more than 20%. Among the remaining variables, 16, 75, and 25 indicators had 0%, 1%-10%, and 10%-20% missing values, respectively. We conducted missing value analysis and according to Little’s Missing Completely at Random (MCAR) test, chi-square was equal to 3346.802 (df = 2855, P < 0.001). Therefore, missing was not completely at random as expected. Since data availability for about 80% of indicators was over 90%, missing values imputation was performed through regressions using all variables as the predictors.

I. Initial regression model
  At this stage, a model was developed to clarify the relations between some important indicators from each group and maternal mortality. In order to create a regression model, 1–2 indicators were selected from each subcategory (Additional file 1) proportional to the number of the indicators in each subcategory and based on the least missing value and the highest correlation with MMR. After developing the linear regression model, high collinearity, i.e. tolerance (T) < 0.2 or variance inflation factor (VIF) > 10, necessitated the elimination of some indicators. As a result, we lost many important indicators like governance indicators and some indicators from most of the groups.
  Moreover, high correlations of some indicators, e.g. mortality and population dynamics and structure indicators, prevented the inclusion of more than 1–2 indicators in either the forward or the stepwise method. It can be explained by the fact that maternal mortality is a mortality indicator having strong correlations with other mortality indexes and life expectancy. Similarly, GFR, which was used for MMR estimation in the model, is highly correlated with young population structure and age-dependency ratios.
  Since many indicators had to be removed from the model, we decided to change our approach, i.e. instead of using single indicators in the regression model, we benefitted from factor analysis (FA) for data reduction and factor construction to be used in a regression analysis.

### Factor analysis (FA)

At the first stage, we ran a FA with principal component analysis (PCA) for factor extraction and Varimax for factor rotation. PCA aimed to extract smaller numbers of more unique global indices as factors instead of single indicators. For easy nomination, we preferred these factors would be more compatible with the World Bank global categorization.

Mortality, population structure, and dynamic indicators were not in included in the FA since they were highly correlated with GFR and MMR (as discussed in the regression model).

Researchers have suggested various methods for selecting the number of factors. Some of these methods are eigenvalues greater than 1, large eigenvalues (without specifying a cut-off point), scree test, examining multiple solutions/interpretability of the solution (including simple structure), a priori number of factors, percentage of variance accounted for, parsimony, parallel, analysis or chi-square test (for maximum likelihood factoring) [20]. However, the recommended cut-off points must be treated flexibly in PCA [21].

All statistical analyses in the current study were conducted with Microsoft Excel 2013 and SPSS for Windows 22.0 (SPSS Inc., Chicago, IL, USA).

## Results

The FA resulted in a nine-factor solution accounting for 61.3% of variance, i.e. 61.3% of the variability of maternal mortality among different countries could be explained by these factors (Additional file 2). Since the extracted factors were not pure enough to be well labeled, we took the following steps:

1. Due to the high number of indicators (38) from different categories loaded to the first factor, we ran a secondary FA on the first factor. After the secondary PCA on the first factor, two new factors were extracted accounting for 76% of variance of the first factor. These new factors were named as 1A and 1B (Table 2).
2. Some of the indicators had relatively high loading on both factors 1 and 2. In order to maximize the orthogonality between the factors [22], ‘improved sanitation facilities, rural (% of rural population with access)’, ‘improved sanitation facilities (% of population with access)’, and ‘school enrollment, secondary (% gross)’ were eliminated from further analysis.
3. In order to ensure better labeling, the indicators were reviewed and refined and some were deleted. For instance, since each communication indicator loaded to different factors, they could not be labeled separately and were thus removed.

**Table 2:** **Factors extracted from FA and indicators loaded to each factor**

**Factor**		**Group (based on World Bank categories)**	**Indicators**
1	1A	Private sector and trade	7 Logistics performance indexes (Overall, competence and quality of logistics services, frequency with which shipments reach consignee within scheduled or expected time, ability to track and trace consignments, Quality of trade and transport-related infrastructure, Efficiency of customs clearance process, Ease of arranging competitively priced shipments), food imports (% of merchandise imports), Private credit bureau coverage (% of adults)
	1B	Governance	Rule of law, political stability and absence of violence/terrorism, control of corruption, voice and Accountability, government effectiveness, regulatory quality, strength of legal rights index
2		Education (input, outcome, efficiency, participation)	Ratio of girls to boys in primary and secondary education (%), Secondary education, pupils (% female), Ratio of female to male secondary enrollment (%)Secondary education, general pupils (% female), Primary education, pupils (% female), Ratio of female to male primary enrollment (%), Primary completion rate, female (% of relevant age group), Primary completion rate, total (% of relevant age group), School enrollment, secondary, female (% gross), School enrollment, primary, female (% net), Primary education, teachers (% female), School enrollment, primary (% net), Pupil-teacher ratio, primary
**3**		Employment and social protection (Labor force structure, Economic Activity)	Employment to population ratio, ages 15–24, female (%), Labor force participation rate for ages 15–24, female (%), Employment to population ratio, 15+, female (%), Labor participation rate, total (% of total population ages 15+), Employment to population ratio, 15+, total (%), Employment to population ratio, ages 15–24, total (%), Labor force participation rate for ages 15–24, total (%), Labor force participation rate, female (% of female population ages 15–64)
**4**		Economic Policy and debt	External balance on goods and services (% of GDP), Gross domestic savings (% of GDP), Gross national expenditure (% of GDP), Final consumption expenditure, etc. (% of GDP), Current account balance (% of GDP), Exports of goods and services (% 6of GDP), External resources for health (% of total expenditure on health)
**5**		Health- service expenditure (services)	Health expenditure, public (% of total health expenditure), Out-of-pocket health expenditure (% of total expenditure on health), Health expenditure, public (% of GDP), Health expenditure, public (% of government expenditure), Health expenditure, private (% of GDP)
**6**		Environment- Agriculture and Production	Crop production index, Food production index
**7**		Education-Efficiency	Repeaters, primary, total (% of total enrollment), Repeaters, primary, female (% of female enrollment)
**8**		Private sector- Private infrastructure	Export volume index, Export value Index
**9**		Economic Policy & debt (goods export and imports)	Goods exports, Goods imports

After the above-mentioned refinements and the final PCA, Kaiser-Meyer-Olkin (KMO) measure of sampling adequacy was calculated as 0.86, i.e. the sample size was enough. The Bartlett’s test of spherocity showed an approximate chi-square of 23380 with a degree of freedom (df) equal to 4371 and a significance level less than 0.05 (0.000). Therefore, the variables were well-correlated in each factor and the whole sample [17].

We used the World Bank terminology for nomination of the extracted components. Table 2 presents the extracted factors and the related indicators loaded to each factor. The definitions of the factors are listed in Additional file 3.

As can be seen in Table 2, most indicators with two figures for females and total were deleted from the results of the FA. Only six indicators finally remained and loaded in the factors: primary completion rate, employment to population ratio 15+ (%), employment to population ratio, ages 15–24 (%), labor force participation rate for ages 15–24, (%), labor force participation rate (%), and repeaters in primary school.

### Multiple regression analysis with extracted factors

In an attempt to investigate the relationships between MMR and the extracted global macrostructural factors, a stepwise multiple linear regression analysis was performed with MMR as the dependent variable and the 10 extracted factors as the predictors (Table 3). Since it was an exploratory analysis without a specific hypothesis about the order of the variables in terms of their probable causal relationships [22], the stepwise method was adopted for including the variables in the multiple regression model.

**Table 3:** **Model summary for stepwise multiple regression model with nine factors**

**Model**	**R** ^**2**^	**Adj. R** ^**2**^	**R** ^**2**^ **change**	**F change**	**Sig. F change**	**Predictors**
1	0.300	0.296	0.300	77.67	<0.001	(Constant), factor score 2
2	0.442	0.436	0.142	45.81	<0.001	(Constant), factor score 2, 1A
3	0.515	0.507	0.073	26.95	<0.001	(Constant), factor score 2, 1A, 1B
4	0.558	0.548	0.043	17.18	<0.001	(Constant), factor score 2, 1A, 1B, 3
5	0.600	0.589	0.043	18.85	<0.001	(Constant), factor score 2, 1A, 1B, 3, 9
6	0.626	0.613	0.025	11.80	<0.001	(Constant), factor score 2, 1A, 1B, 3, 9, 7
7	0.649	0.634	0.023	11.45	0.001	(Constant), factor score 2, 1A, 1B, 3, 9, 7, 4
8	0.661	0.645	0.012	6.34	0.013	(Constant), factor score 2, 1A, 1B, 3, 9, 7, 4, 6
9	0.671	0.654	0.010	5.27	0.023	(Constant), factor score 2, 1A, 1B, 3, 9, 7, 4, 6, 5

The excluded variable in this model was factor score 8 (export value index and export volume index) of Table 2, with ln B (natural logarithm) = −0.41, t = −0.80, and P = 0.42. All remaining factors had significant F changes. Consequently, the effect of each factor entered in the model was significant and probability that the results had happened by chance was less than 0.05 for all factors.

Factor scores 2 (education), 1A (private sector and trade), and 1B (governance) were the first factors to enter the regression equation and had the highest correlation with global maternal mortality. These three factors accounted for 52% of maternal mortality variation between countries. An interesting finding showed that heath expenditure, as the only ecological health indicator in this model, was the last factor to enter the model and was responsible for only 10% of variance. The R^2^ of the final model (67.1%) represented the variance of MMR which was associated with the predictive factors in the model. Adjusted R^2^, a more conservative indicator for variance which estimates the expected shrinkage if the model is applied to another sample [17], was as high as 65.4% in this study. Table 4 summarizes the coefficients of the final model (the constant and nine factors).

**Table 4:** **Coefficients of the final regression model with MMR 2010 as the dependent variable**

**Model**	**Unstandardized coefficients**		**Standardized coefficients**	**t**	**P-value**	**Collinearity statistics**	
	**B**	**Std. error**	**Beta**			**Tolerance**	**VIF**
(Constant)	171.04	9.71	-	17.63	0.001	-	-
Factor score 2 (education input, outcome, efficiency, participation)	−98.54	10.38	−0.44	−9.50	0.001	0.88	1.14
Factor score 1 A (private sec & trade)	−70.45	10.42	−0.32	−6.76	0.001	0.87	1.15
Factor score 1 B (governance)	−62.51	11.35	−0.28	−5.51	0.001	0.74	1.36
Factor score 3 (employment & labor)	46.90	9.81	0.21	4.78	0.001	0.98	1.02
Factor score 9 (goods import & export)	−46.77	9.76	−0.21	−4.79	0.001	0.99	1.01
Factor score 7 (education efficiency-repeaters)	35.67	9.84	0.16	3.62	0.001	0.97	1.02
Factor score 4 (economic policy & debt)	−32.30	10.35	−0.15	−3.12	0.002	0.89	1.13
Factor score 6 (agriculture: food and crop production)	26.06	9.81	0.12	2.66	0.009	0.98	1.02
Factor score 5 (health service expenditure)	−24.25	10.56	−0.11	−2.30	0.023	0.85	1.18

The results of the last regression model showed no collinearity among the nine loaded factors in the model, i.e. these extracted factors had not significant correlations with each other. Regression coefficients are generally calculated to estimate the average change in the dependent variable for one unit of change in an independent (predictor) variable while maintaining other predictors in the model constant [23]. On the other hand, standardized coefficients make unstandardized coefficients comparable in terms of measurement unit based on z scores with a mean of 0 and a standard deviation (SD) of 1 [23,24].

The *Std. Error* column in Table 4 includes the standard errors of the regression coefficients. In fact, 95% confidence interval (CI) of B can be made by B ± 2 Std. Error. Moreover, *t* is a measure of the likelihood that the actual value of the parameter is not zero. In other words, SPSS tests the significance of each predictor in the equation [17]. The large absolute value of this statistic is in favor of rejecting null hypothesis. Therefore, nine out of 10 factors were statistically significant in the final model.

As we only entered the factors, not the indicators, in the described regression analysis, it was difficult to present their coefficients. For example, if education were a unique indicator with a specific scale, we could have concluded that one unit change in the global education could decrease 98.5 maternal deaths in 100,000 live births at the global level. However, since education was a factor comprising different indicators (Table 2), such a conclusion could not be made. In order to place input variables on a common scale, each numeric variable is generally divided by its SD. As explained earlier, standardizing both the predictors and the response would lead to a standard model based on z scores with a mean of 0 and SD of 1 [23,24]. Hence, in the previous example, one SD increase in global education decreased global maternal mortality by 0.441 of its SD. This method made the effects of all predictors comparable.

As seen, all obtained coefficients, except for employment and labor, education efficiency (repeaters) and agriculture (crop and food production), were negative, i.e. an increase in each factor decreased MMR.

Leverage is a term used in regression analysis to identify the observations which are far from the corresponding average predictor values [25] and to check the extreme values. In cases of data points with high leverage, Cook’s distance would be an important diagnostic tool for detecting the influential individual or groups of observations for cross-sectional data [26]. Cook’s distance combines information from the studentized residuals and the variances of the residuals and predicted values [27]. Large values of Cook’s distance signify unusual observations. Values greater than 1 require careful checking and those greater than 4 are potentially serious outliers. Since a point with leverage greater than (2 k + 2)/n, where k is the number of predictors and n is the number of observations, should be carefully examined [28], (2 * 10 + 2)/179 = 0.1229 was the cut-off point in our model. None of the factors in the regression model had a leverage higher than the mentioned cut-off point. Moreover, a Cook’s distance larger than 1 was not seen in any case.

## Discussion

### Education

The highest correlations in this study were observed in case of the education group of the indicators with two factors in the regression model. The first one, including input, outcome, efficiency, and participation indicators (based on the World Bank classification), had a negative regression coefficient in the model. As explained earlier, one SD increase in global education associates with decrease in global maternal mortality by 0.44 SD. Conversely, the seventh factor, i.e. education efficiency, had a positive regression coefficient. Since this factor comprised indicators related to primary school repeaters, one SD decrease in the percentage of global repeaters (increasing education efficiency) associates with decrease in global maternal mortality by 0.16 of the global MMR SD. Although previous studies have addressed the effects of education, especially women education, on MMR [29-37], not many researchers have backed up this hypothesis by statistical correlations. While the sixth loaded factor in the present study was a separate factor, it could be discussed under education category. The World Bank classification (Additional file 1) indicates that repeaters can interpret the efficiency of education, i.e. repeaters reaching one-fifth of the students in some countries with high MMR reveal the insufficiency of the educational system and wasting of the available resources. However, health literature has scarcely differentiated between various aspects of education such as input, outcome, participation, and efficiency. Further research is hence required to compare the effects of each aspect of education on not only MMR, but also other health-related indicators.

### Private sector and trade

The second factor included in our regression model, i.e. private sector and trade, consisted of seven indicators related to logistic performance. As it had a negative regression coefficient, one SD improvement in global logistic performance and trade associates with decrease in global maternal mortality by 0.32 SD. The World Bank (Additional file 3) has defined logistics as the activities, e.g. transportation, warehousing, packaging, and material handling that manage the flows of goods, cash, and information between the point of supply and the point of demand. Inefficient logistics structure imposes additional time and financial costs and exerts negative effects on the competitiveness of both enterprises and countries [38,39]. The logistics performance index reflects perceptions of a country’s logistics based on the efficiency of customs clearance process, quality of trade- and transport-related infrastructures, ease of competitively-priced shipment arrangements, quality of logistics services, ability to track and trace consignments, and frequency with which shipments reach the consignee within the scheduled time [16]. Despite the scarcity of studies on the relation between health and logistic performance indicators, social indicators such as expected years of schooling and gross national income have been surprisingly shown to be more related with logistics performance than economic indicators in 26 members of the Organization for Economic Cooperation and Development (OECD) [38].

### Governance

The third factor can be expressed as dimensions of governance which had a negative regression coefficient. In fact, one SD increase in global governance associates with decrease in global MMR by 0.28 SD. Governance can be described as a set of traditions and conventions that determines the practice of authority in a particular country. It comprises not only the processes through which governments are selected, held accountable, monitored, and replaced, but also the capacity of governments to efficiently manage resources and formulate, implement, and enforce appropriate policies and regulations. In addition, governance regulates the level of respect received by the citizens and the state for conventions and laws that govern the economic and social interactions in the community [40].

Muldoon underscored the direct effects of government corruption on child and maternal mortality [41]. Apparently, improved governance has large causal effects on better development outcomes [40]. Consequently, differences in the efficacy of public spending on child mortality rate reduction can be attributed to the quality of governance in various countries. Likewise, public spending on primary education can more effectively enhance primary education achievements in countries with better governance. Generally, public spending has almost no impact on health and education outcomes in poorly governed countries [42]. On the other hand, the positive impacts of appropriate governance on income and quality of the health care sector can promote public health [43]. Studies have shown that while absolute income is the most important determinant of health in less developed countries, governance plays the most critical role in more developed countries [44]. Nevertheless, despite the significance of governance in human resources for health (HRH) policy development and implementation, a review concluded that the term ‘governance’ has not been frequently used in the recent HRH literature [45].

### Employment and labor workforce

Another important factor in the current regression model was employment and labor workforce structure. Surprisingly, maternal mortality was found to be positively related with employment and labor indicators (standardized coefficient = 0.21). Research has shown a negative relationship between unemployment and health [46] which can be affected by welfare state and social protection regime. As such a negative relationship could be caused by lower than average wage replacement rates of unemployed women [46], policies which widen the educational gaps or influence employment opportunities and social gradient would impose adverse effects on health equity and other social outcomes [47].

Further analysis of our findings indicated that all components (indicators) of employment and labor workforce had positive bivariate correlations with MMR. Additional probing suggested the results to be based on a clear ecological bias caused by between-country variability of employment and wage conditions. This, however, has to be explored in a separate manuscript in the future.

### Economic policy and debt

Under this category of the World Bank classification, the fifth and seventh factors, both with negative standardized regression coefficients (0.21 and 0.15, respectively) were entered into the model. These factors consisted of indicators related to goods and services, domestic savings and expenditure, and national current accounts (Table 2) (Additional file 3). The harmful effects of economic dependency, especially multinational corporate investment, on maternal mortality have been well documented. Such effects are known to be mediated by the negative impacts of economic dependency on economic growth and women’s status [48]. On the other hand, some researchers have underscored the significance of technical and financial support from a developing country’s international partners, e.g. bilateral donors, UN agencies, and regional development banks, in the implementation of its development strategies, particularly after the global economic crisis. Consequently, the development of countries strongly depends on the governments’ economic policies for distribution of the aid resources and efficient public investment management [49-52]. It was interesting that ‘external resources for health (% of total expenditure on health)’ was loaded to this factor.

The global economy can in fact influence the achievement of MDGs by facilitating economic growth in particular countries. It can also affect the progress of MDGs through the modification of financial flows to decrease difficulties due to budget constraint [53]. Domestic growth provides private incentives and public resources for sustainable progress in non-income MDGs.

### Food and crop production

In contrast to our baseline hypothesis, we found maternal mortality to be positively correlated with food and crop production indexes (standardized coefficient = 0.12). In the absence of clear evidence to confirm the relation between maternal mortality and food and crop production, the existing data suggests food availability as a determinant of health status. According to previous studies, a mere focus on health service provision, family planning programs, and emergency aids without attention to socioeconomic and environmental aspects (such as food production) may be of little benefit in the current health status of vulnerable areas like Sub-Saharan Africa the region [54]. Meanwhile, practical measures on the structural drivers of food availability, accessibility, and acceptability are warranted to address not only the effects of food price during economic crisis on health [5], but also nutrition inequality as a determinant of health at both global and national levels [55]. The ecologic bias of this relation should be further clarified by investigating intra-country variability in other indicators such as food availability and distribution and trade policies.

### Health expenditure

The lowest absolute value of regression coefficients among other global factors in our regression model belonged to health expenditure. In other words, one SD increase in global health expenditure was associated with 0.11 SD decrease in global maternal mortality. Assessment of the indicators composing this factor and their bivariate correlations with MMR suggested greater share of governmental health expenditure to be negatively related with maternal mortality. In contrast, private sector share and out-of-pocket health expenditure showed a positive correlation. Since appropriate government financing can ensure better access to some essential maternal health services, greater absolute levels of health expenditure will be required for developing countries in order to achieve MDG on maternal mortality [56]. Total health expenditure varies between around 2%-3% of gross domestic product (GDP) in low-income countries (< $1000 per capita) to 8%-9% of GDP in high-income countries (> $7000 per capita). Contrary to our expectation, poor countries and communities, i.e. groups with the greatest need for protection from financial catastrophe, receive the least level of support in the form of prepayment and risk-sharing. While the mean out-of-pocket expenditure in low-income countries is as high as 20%-80% of the total expenditure, the rates drops sharply and the variation narrows in high-income countries. In other words, increased income is associated with greater public financing and higher share of GDP and health from total public expenditure [57]. As the existing degrees of public-health expenditure in many developing countries are far different from the targeted values [58], revising national health policies to address the current inequalities, promote a long-term perspective plan, and concentrate on a paradigm shift from the current ‘biomedical model’ to a ‘sociocultural model’ is essential to tackle the numerous health problems in these countries [59].

In a book entitled as ‘Equity, social determinants and public health programs’ published by WHO [36], the authors discussed that the first obvious social determinant of a woman’s chance of having a skilled birth attendant was spending on health. In fact, greater government contribution in health financing and higher levels of health spending would improve maternal health services including the presence of skilled birth attendants. In the same book, the logarithm of public health expenditure was reported to be linearly related with access to skilled attendance at birth. Moreover, the percentage of births with skilled attendance was found to be negatively correlated with private health and out-of-pocket health expenditure (both as proportions of total health expenditure). The authors explained that the effect of skilled birth attendance on maternal mortality depended on the cause of maternal complications, quality of care, administration of appropriate pharmaceuticals, and the presence of a proper referral system [36].

### Study limitations

Since the analyses were performed on cross-sectional data, no causal relationships could be examined. However, it can be inferred that low education can lead to higher maternal mortality (the opposite cannot be true). On the other hand, since we extracted data from the existing global datasets, many important groups of indicators, e.g. gender and inequality, were removed due to the high level of missing values. Furthermore, considering the fact that geographic aggregation of data can influence the conclusions about the nature and extent of differences across populations in various geographic areas. So, the level of inference in this study should just be the global level and inter-country variability should be considered to inform priority setting in a country. Furthermore, we did not check the normal distribution of all indicators due to their high number (n = 439). Moreover, we took into consideration that indicator transformation will make the results hard to be presented and discussed because of using factors in the regression analysis comprised of simple and transformed indicators. We believed that as a result of large sample size and the Law of Large Numbers, the distributions tended to be normal and Central Limit Theorem was considerable.

## Conclusion

Evaluating the role of policies in the achievement of different MDGs can shed light on the existing difficulties and obstacles and facilitate the modification of the present public policies to efficiently meet these targets [60]. According to previous studies, the most successful interventions essentially tackle a particular problem by combining a wide range of intersectoral and upstream approaches with downstream interventions [61].

Upon the establishment of a relationship between better distribution of economic and social resources and health indicators, Navarro suggested more appropriate redistribution of resources, e.g. labor market resources (such as employment), welfare state resources (such as healthcare coverage, public health expenditures, education, and family supportive services), social transfer resources, cultural resources (such as civil associations), and political resources (such as the distribution of power), to be critical to the improvement of health indicators [62].

Some researchers believe that some socioeconomic, environmental, and political factors are poorly discussed in the health literature. These factors include environmental modifications, adoption, incorporation, and enforcement of human rights conventions within the legal structure, regressive/progressive structure of taxes, minimum wage guarantees and their ratio to the overall wage structures, government corruption, and representativeness of legislatures relative to sociodemographic population distributions [63]. This paper sought to illuminate the association of a group of these indicators with global maternal mortality.

Due to the obvious scarceness of the available health resources and the role of politics, values, and resources in decision-making about their allocation [64], the UN Millennium Project has recommended that every developing country with extreme poverty should adopt and implement an ambitious national development strategy to achieve the MDGs [49].

As explained earlier, evidence on policy interventions to reduce maternal mortality is not strong. In other words, while some studies have only investigated individual determinants and medical interventions, in their efforts to examine ecological factors, others have mostly focused on outcome indicators of the same distal policies that influenced maternal death.

Reducing maternal mortality is a critical and challenging MDG. Maternal death is believed to be affected by not only the properties of the health system and service delivery, but also several other factors outside of the health system. Nevertheless, robust health information systems and health statistics are necessary to implement planning and strategic decision-making programs, monitor the progress toward the targets, and assess the feasibility of various strategies [65].

A clear analysis of both proximal and distal determinants of a specific situation, e.g. maternal mortality, is indispensable to its improvement. Since ethical principles are capable of motivating and holding global and national actors accountable for achieving common global goals, international and national responses to health disparities must be rooted in core ethical values about health and its distribution [66]. Similarly, political will, increased funding, and social support for women’s health can largely contribute to decreased maternal mortality [67]. Efforts to lower maternal mortality without basic maternal health services are unlikely to become available without pro-poor health policies and will thus fail [68]. Moreover, re-allocation of national resources to development, especially health and education, is essential [58]. Since all MDGs are inter-correlated, measures to expand maternal health service utilization can be accelerated by parallel investments in programs aimed at poverty eradication (MDG 1), universal primary education (MDG 2), and women’s empowerment (MDG 3) [37]. Within the health sector, programs can shift human and financial resources to both reach underserved populations and increase overall availability of services. In parallel, policies can improve the accessibility and acceptability of services by protecting reproductive rights and expanding knowledge of sexual and reproductive health. Furthermore, communities can reduce gender inequity by ensuring equal access to educational and financial opportunities for both men and women [36].

According to the results of the current research, factors affecting maternal mortality are beyond the individual level. They can in fact be influenced by other countries and even international institutions. More precisely speaking, trade, debt, import and export, political stability, and strength of legal rights can be determined by factors beyond the borders of the countries or territories and even by the global situation and challenges. The pathways for their effects on maternal mortality could be through the effect on country development.

In summary, vision is the most critical issue in achieving MDGs. Although countries have clearly stated their vision upon their registration for MDG-5, such statements would be meaningless in the absence of a clear strategy for their accomplishment [64]. Therefore, in order to design effective multilevel strategies, global approaches should be adopted and the existing situations in each country have to be analyzed. In addition, health policymakers need to be aware of the potential of macrostructural indicators such as governance, education, economic policies, and sociocultural policies to limit or enhance health opportunities for different groups in the population. These indicators can enlighten the way for sustainable development in Post-2015 Development Agenda. We believe that a new agenda for health researchers is to provide both health and non-health policymakers with interdisciplinary information to signal them about the policies which may undermine the efforts to promote health. In other words, some of the health indicators, e.g. maternal mortality, are not achievable without multi-faceted development and a comprehensive approach toward health policies at national and international levels.

## Additional files

Additional file 1:
**List of the selected relevant indicators with bivariate correlation more than 0.2 with MMR, missing values less than 20% and P-value less than 0.05 by category.**
Additional file 2:
**Total Variance explained by the factors in the first Factor Analysis.**
Additional file 3:
**Indicator definitions based on World Bank.**

## Abbreviations

FA: Factor analysis
GDP: Gross domestic product per capita based on purchasing power parity conversion
GFR: Gross fertility rate
HRH: Human resources for health
MAR: Missing at Random
MCAR: Missing Completely at Random
MDGs: Millennium Development Goals
MMR: Maternal mortality ratio
OECD: The Organization for Economic Cooperation and Development
RAMOS: Reproductive age mortality studies
SAB: Presence of a skilled attendant at birth as a proportion of total birth
SD: Standard deviation
UT: University of Tehran
VIF: Variance inflation factor
WHO: World Health Organization
