# Supplementary material for: Maternal mortality: a cross-sectional study in global health
Source: Global Health. 2015 Feb 12;11:4. doi: 10.1186/s12992-015-0087-y (PMC4353673; doi:10.1186/s12992-015-0087-y)
Supplement: Additional file 1: — List of the selected relevant indicators with bivariate correlation more than 0.2 with MMR, missing values less than 20% and P-value less than 0.05 by category. [file 12992_2015_87_MOESM1_ESM.docx]

**Additional File 1**

**List of the selected relevant indicators with bivariate correlation more than 0.2 with MMR, missing values less than 20% and significance level less than 0.05 by category**

We used the World Bank categories in World Development Indicator Database (<http://databank.worldbank.org/data>)

| **Category** | **subcategory** | **Number of indicators** | **Total** |
| --- | --- | --- | --- |
| Economic Policy and debt | current account balance | 4 | 14 |
|  | National accounts | 10 |  |
| Education | Input | 2 | 21 |
|  | Outcome | 2 |  |
|  | Efficiency | 4 |  |
|  | Participation | 13 |  |
| Health | Disease Prevention | 4 | 26 |
|  | Health services | 10 |  |
|  | Mortality | 5 |  |
|  | population structure | 1 |  |
|  | Population dynamics | 3 |  |
|  | Reproductive health | 1 |  |
|  | nutrition | 2 |  |
| Infrastructure | Communication | 6 | 6 |
| Private sector and Trade | Business Environment | 6 | 24 |
|  | business start up | 1 |  |
|  | export and imports | 3 |  |
|  | Private infrastructure Investment | 14 |  |
| employment & social protection | Labor force structure | 4 | 8 |
|  | Economic activity | 3 |  |
|  | Migration | 1 |  |
| Governance | - | 6 | 6 |
| Environment | Agriculture and Production | 11 | 12 |
| Public sector | Conflict and fragility | 1 |  |
| Total | | | 116 |
