# Supplementary material for: Maternal mortality: a cross-sectional study in global health
Source: Global Health. 2015 Feb 12;11:4. doi: 10.1186/s12992-015-0087-y (PMC4353673; doi:10.1186/s12992-015-0087-y)
Supplement: Additional file 2: — Total Variance explained by the factors in the first Factor Analysis. [file 12992_2015_87_MOESM2_ESM.docx]

**Additional File 2**

Total Variance explained by the extracted factors in the first Factor Analysis

| **Factor** | **Initial Eigenvalues** | | **Rotation Sum of Squared Loading** | | |
| --- | --- | --- | --- | --- | --- |
|  | **Total** | **% of variance** | **Total** | **% of variance** | **Cumulative %** |
| 1 | 30.0 | 31.9 | 20.8 | 22.1 | 22.1 |
| 2 | 8.4 | 8.9 | 9.5 | 10.1 | 32.2 |
| 3 | 5.8 | 6.2 | 8.0 | 8.5 | 40.7 |
| 4 | 5.6 | 6.0 | 6.4 | 6.8 | 47.5 |
| 5 | 3.5 | 3.7 | 3.3 | 3.5 | 51.0 |
| 6 | 2.6 | 2.8 | 2.8 | 3.0 | 54.0 |
| 7 | 2.4 | 2.6 | 2.3 | 2.5 | 56.5 |
| 8 | 2.1 | 2.2 | 2.3 | 2.5 | 59.0 |
| 9 | 1.9 | 2.0 | 2.2 | 2.3 | 61.3 |
