# Supplementary material for: Maternal mortality: a cross-sectional study in global health
Source: Global Health. 2015 Feb 12;11:4. doi: 10.1186/s12992-015-0087-y (PMC4353673; doi:10.1186/s12992-015-0087-y)
Supplement: Additional file 3: — Indicator definitions based on World Bank. [file 12992_2015_87_MOESM3_ESM.docx]

# **Additional File 3: Indicator definitions based on World Bank**

- **Control of Corruption: Estimate**: Control of Corruption captures perceptions of the extent to which public power is exercised for private gain, including both petty and grand forms of corruption, as well as "capture" of the state by elites and private interests. Estimate gives the country's score on the aggregate indicator, in units of a standard normal distribution, i.e. ranging from approximately -2.5 to 2.5.
- **Crop production index (2004-2006 = 100):** Crop production index shows agricultural production for each year relative to the base period 2004-2006. It includes all crops except fodder crops. Regional and income group aggregates for the FAO's production indexes are calculated from the underlying values in international dollars, normalized to the base period 2004-2006.
- **Export value index (2000 = 100):** Export values are the current value of exports (f.o.b.) converted to U.S. dollars and expressed as a percentage of the average for the base period (2000). UNCTAD's export value indexes are reported for most economies. For selected economies for which UNCTAD does not publish data, the export value indexes are derived from export volume indexes (line 72) and corresponding unit value indexes of exports (line 74) in the IMF's International Financial Statistics.
- **Export volume index (2000 = 100)**: Export volume indexes are derived from UNCTAD's volume index series and are the ratio of the export value indexes to the corresponding unit value indexes. Unit value indexes are based on data reported by countries that demonstrate consistency under UNCTAD quality controls, supplemented by UNCTAD’s estimates using the previous year’s trade values at the Standard International Trade Classification three-digit level as weights. To improve data coverage, especially for the latest periods, UNCTAD constructs a set of average prices indexes at the three-digit product classification of the Standard International Trade Classification revision 3 using UNCTAD’s Commodity Price Statistics, interna­tional and national sources, and UNCTAD secretariat estimates and calculates unit value indexes at the country level using the current year’s trade values as weights. For economies for which UNCTAD does not publish data, the export volume indexes (lines 72) in the IMF's International Financial Statistics are used.
- **External balance on goods and services (% of GDP):** External balance on goods and services (formerly resource balance) equals exports of goods and services minus imports of goods and services (previously nonfactor services).
- **Food imports (% of merchandise imports):** Food comprises the commodities in SITC sections 0 (food and live animals), 1 (beverages and tobacco), and 4 (animal and vegetable oils and fats) and SITC division 22 (oil seeds, oil nuts, and oil kernels).
- GDP growth (annual %): Annual percentage growth rate of GDP at market prices based on constant local currency. Aggregates are based on constant 2000 U.S. dollars. GDP is the sum of gross value added by all resident producers in the economy plus any product taxes and minus any subsidies not included in the value of the products. It is calculated without making deductions for depreciation of fabricated assets or for depletion and degradation of natural resources.

| - **Goods exports (BoP, current US$)**: Goods exports refer to all movable goods (including nonmonetary gold) involved in a change of ownership from residents to nonresidents. The category includes goods previously included in services: goods received or sent for processing and their subsequent export or import in the form of processed goods, repairs on goods, and goods procured in ports by carriers. Data are in current U.S. dollars. - **Goods imports (BoP, current US$):** Goods imports refer to all movable goods (including nonmonetary gold) involved in a change of ownership from nonresidents to residents. The category includes goods previously included in services: goods received or sent for processing and their subsequent export or import in the form of processed goods, repairs on goods, and goods procured in ports by carriers. - **Government Effectiveness: Estimate**: captures perceptions of the quality of public services, the quality of the civil service and the degree of its independence from political pressures, the quality of policy formulation and implementation, and the credibility of the government's commitment to such policies. Estimate gives the country's score on the aggregate indicator, in units of a standard normal distribution, i.e. ranging from approximately -2.5 to 2.5. - **Food production index:** Food production index covers food crops that are considered edible and that contain nutrients. Coffee and tea are excluded because, although edible, they have no nutritive value. - **Health expenditure, private (% of GDP):** Private health expenditure includes direct household (out-of-pocket) spending, private insurance, charitable donations, and direct service payments by private corporations. - **Health expenditure, public (% of government expenditure):** Public health expenditure consists of recurrent and capital spending from government (central and local) budgets, external borrowings and grants (including donations from international agencies and nongovernmental organizations), and social (or compulsory) health insurance funds. - **Logistics performance index:** Ability to track and trace consignments (1=low to 5=high): Logistics professionals' perception of the ability to track and trace consignments when shipping to the country, on a rating ranging from 1 (very low) to 5 (very high). Scores are averaged across all respondents. |
| --- |
| - **Logistics performance index: Competence and quality of logistics services (1=low to 5=high):** Logistics professionals' perception of country's overall level of competence and quality of logistics services (e.g. transport operators, customs brokers), on a rating ranging from 1 (very low) to 5 (very high). Scores are averaged across all respondents. |
| - **Logistics performance index: Ease of arranging competitively priced shipments (1=low to 5=high):** Logistics professionals' perception of the ease of arranging competitively priced shipments to a country, on a rating ranging from 1 (very difficult) to 5 (very easy). Scores are averaged across all respondents. |
| - **Logistics performance index: Efficiency of customs clearance process (1=low to 5=high):** Logistics professionals' perception of the efficiency of country's customs clearance processes (i.e. speed, simplicity and predictability of formalities), on a rating ranging from 1 (very low) to 5 (very high). Scores are averaged across all respondents. |
| - **Logistics performance index: Frequency with which shipments reach consignee within scheduled or expected time (1=low to 5=high):** Logistics professionals' perception of how often the shipments to assessed country reach the consignee within the scheduled or expected delivery time, on a rating ranging from 1 (hardly ever) to 5 (nearly always). Scores are averaged across all respondents. |
| - **Logistics performance index: Overall (1=low to 5=high):** Logistics Performance Index overall score reflects perceptions of a country's logistics based on efficiency of customs clearance process, quality of trade- and transport-related infrastructure, ease of arranging competitively priced shipments, quality of logistics services, ability to track and trace consignments, and frequency with which shipments reach the consignee within the scheduled time. The index ranges from 1 to 5, with a higher score representing better performance. |
| - **Logistics performance index: Quality of trade and transport-related infrastructure (1=low to 5=high):** Logistics professionals' perception of country's quality of trade and transport related infrastructure (e.g. ports, railroads, roads, information technology), on a rating ranging from 1 (very low) to 5 (very high). Scores are averaged across all respondents. - **Out of pocket expenditure:**  is any direct outlay by households, including gratuities and in-kind payments, to health practitioners and suppliers of pharmaceuticals, therapeutic appliances, and other goods and services whose primary intent is to contribute to the restoration or enhancement of the health status of individuals or population groups. It is a part of private health expenditure. - **Political Stability and Absence of Violence/Terrorism: Estimate:** Political Stability and Absence of Violence/Terrorism captures perceptions of the likelihood that the government will be destabilized or overthrown by unconstitutional or violent means, including politically-motivated violence and terrorism. Estimate gives the country's score on the aggregate indicator, in units of a standard normal distribution, i.e. ranging from approximately -2.5 to 2.5. |

- **Private credit bureau coverage (% of adults**): Private credit bureau coverage reports the number of individuals or firms listed by a private credit bureau with current information on repayment history, unpaid debts, or credit outstanding. The number is expressed as a percentage of the adult population.
- **Regulatory Quality: Estimate:** Regulatory Quality captures perceptions of the ability of the government to formulate and implement sound policies and regulations that permit and promote private sector development. Estimate gives the country's score on the aggregate indicator, in units of a standard normal distribution, i.e. ranging from approximately -2.5 to 2.5.
- **Rule of Law: Estimate:** Rule of Law captures perceptions of the extent to which agents have confidence in and abide by the rules of society, and in particular the quality of contract enforcement, property rights, the police, and the courts, as well as the likelihood of crime and violence. Estimate gives the country's score on the aggregate indicator, in units of a standard normal distribution, i.e. ranging from approximately -2.5 to 2.5.
- **Strength of legal rights index (0=weak to 10=strong):** Strength of legal rights index measures the degree to which collateral and bankruptcy laws protect the rights of borrowers and lenders and thus facilitate lending. The index ranges from 0 to 10, with higher scores indicating that these laws are better designed to expand access to credit.
- **Voice and Accountability:** Estimate: Voice and Accountability captures perceptions of the extent to which a country's citizens are able to participate in selecting their government, as well as freedom of expression, freedom of association, and a free media. Estimate gives the country's score on the aggregate indicator, in units of a standard normal distribution, i.e. ranging from approximately -2.5 to 2.5.
